# Sequence variants from whole genome sequencing a large group of Icelanders

**DOI:** 10.1038/sdata.2015.11

**Published:** 2015-03-25

**Authors:** Daniel F Gudbjartsson, Patrick Sulem, Hannes Helgason, Arnaldur Gylfason, Sigurjon A Gudjonsson, Florian Zink, Asmundur Oddson, Gisli Magnusson, Bjarni V Halldorsson, Eirikur Hjartarson, Gunnar Th. Sigurdsson, Augustine Kong, Agnar Helgason, Gisli Masson, Olafur Th. Magnusson, Unnur Thorsteinsdottir, Kari Stefansson

**Affiliations:** 1 deCODE genetics/Amgen, Inc., Reykjavik 101, Iceland; 2 School of Engineering and Natural Sciences, University of Iceland, Reykjavik 101, Iceland; 3 Institute of Biomedical and Neural Engineering, Reykjavík University, Reykjavík 101, Iceland; 4 Department of Anthropology, University of Iceland, Reykjavik 101, Iceland; 5 Faculty of Medicine, University of Iceland, Reykjavik 101, Iceland

**Keywords:** Next-generation sequencing, Genetic variation, DNA sequencing, Genetic markers

## Abstract

We have accumulated considerable data on the genetic makeup of the Icelandic population by sequencing the whole genomes of 2,636 Icelanders to depth of at least 10X and by chip genotyping 101,584 more. The sequencing was done with Illumina technology. The median sequencing depth was 20X and 909 individuals were sequenced to a depth of at least 30X. We found 20 million single nucleotide polymorphisms (SNPs) and 1.5 million insertions/deletions (indels) that passed stringent quality control. Almost all the common SNPs (derived allele frequency (DAF) over 2%) that we identified in Iceland have been observed by either dbSNP (build 137) or the Exome Sequencing Project (ESP) while only 60 and 20% of rare (DAF<0.5%) SNPs and indels in coding regions, the most heavily studied parts of the genome, have been observed in the public databases. Features of our variant data, such as the transition/transversion ratio and the length distribution of indels, are similar to published reports.

## Background & Summary

Genome-wide association scans were initially based on 300–600 k SNP chip genotyping arrays designed based on the HapMap dataset^1^. The HapMap project focused primarily on common variants (MAF>5%) and methods were subsequently developed to accurately impute 2.5 million HapMap phase 2 SNPs into such chip data^2^. This has led to the discovery of a plethora of associations between common sequence variants and human diseases and traits^3^.

Large scale whole genomic sequencing has allowed the detection of rare sequence variants that range in effect from causing diseases to modifying complex disease risk—variants that would recently either not have been observed or could not be tested for association with disease on a sufficiently large scale. Several large sequencing projects are ongoing such as the 1000 Genomes project^4^, the Exome sequencing project (ESP)^5,6^ and the GoNL project^7^.

**Figure 1:**
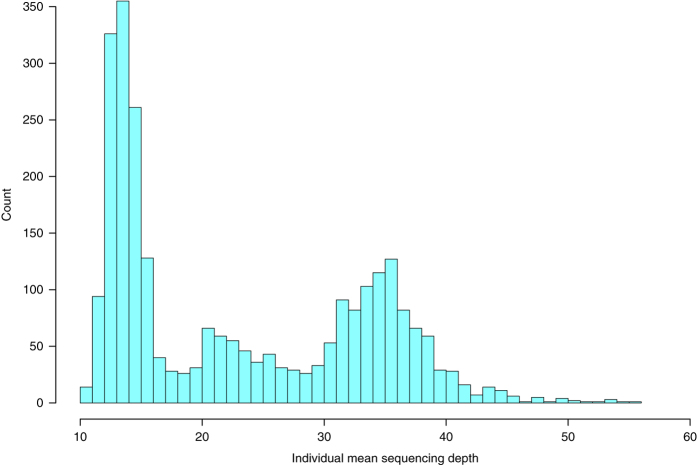
Sequencing depth by individual. A histogram of the individual mean sequencing depth of the 2,636 whole-genome sequenced Icelanders. Figure reproduced from Supplementary Fig. 1 of ref. 13.

**Figure 2:**
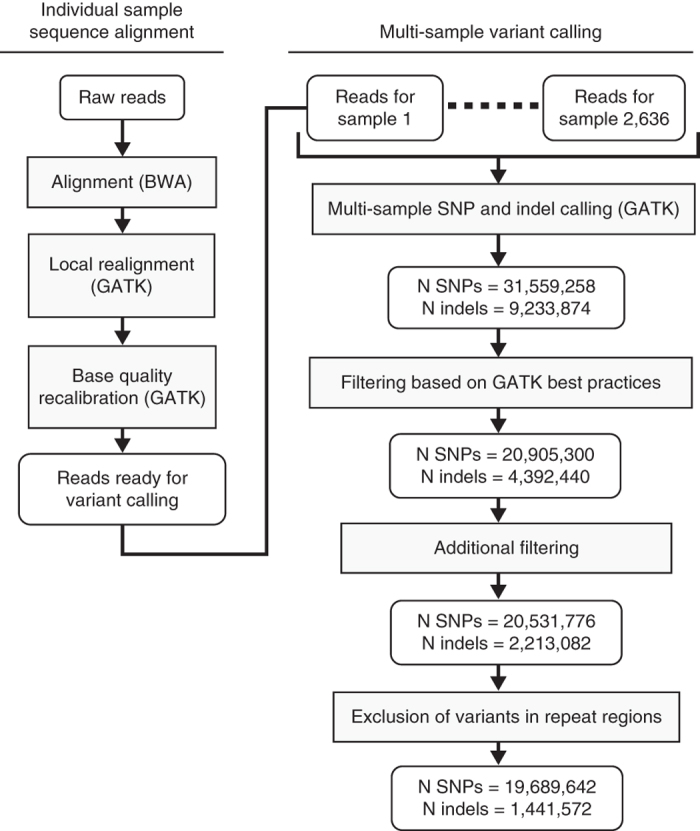
Overview of sequence alignment and variant calling. Figure reproduced from Supplementary Fig. 2 of ref. 13.

We have sequenced the whole genomes of 2,636 Icelanders using Illumina technology. The individuals were selected for sequencing based on having a wide range of phenotypes (Tables 1 and 2). The sequencing was done to a mean depth of at least 10X (median 20X), including 909 to a mean depth of at least 30X (Fig. 1). For individuals with an average depth of at least 10X, a coverage of at least 1X was achieved for 2.72 Gb and of 10X or more for 2.70 Gb. For individuals with an average depth of at least 30X, a coverage of at least 30X was achieved for 2.35 Gb. A total of 20 million autosomal SNPs and 1.5 million indels, up to a length of 60 base-pairs (bp), were identified and their genotypes called for all samples simultaneously using the Genome Analysis Toolkit (GATK version 2.3.9, Fig. 2)^8^. We used information about haplotype sharing, taking advantage of the fact that all the sequenced individuals had also been chip-typed and long range phased to improve variant genotyping^9^.

The effect of the sequence variants on the 19,135 protein coding genes in the RefSeq database^10^ was annotated using the Variant Effect Predictor (VEP)^11^. The VEP predicts the consequence of each sequence variant on all neighboring RefSeq genes based on a set of 35 consequence terms defined by the Sequence Ontology (SO) (Table 3)^12^. We grouped sequence variants into four categories, in order of decreasing severity: i) LoF, loss of function including stop gained/lost variants, frameshift indels, donor/acceptor splice and initiator codon variants (*N*=6,795), ii) MODERATE, including missense variants, inframe indels and splice region variants (*N*=125,542), iii) LOW, including synonymous variants 3′ and 5′ UTR variants (289,166); and iv) OTHER, including deep intronic and inter-genic variants (*N*=20,709,711). We find that the fraction of variants with a minor allele frequency (MAF) below 0.1% (corresponding to five or fewer copies of the minor allele in our data), is 61.6, 46.4, 37.5 and 36.0% in the LoF, MODERATE, LOW and OTHER categories, respectively (Table 4).

## Methods

These methods are an expanded version of the descriptions contained in Gudbjartsson *et al.*^13^

### The Icelandic study population

This study is based on whole-genome sequence data from the whole blood of 2,636 Icelanders participating in various disease projects at deCODE genetics (Tables 1 and 2). In addition, a total of 104,220 Icelanders have been genotyped using Illumina SNP chips.

All participating individuals, or their guardians, gave their informed consent before blood samples were drawn. The family history of participants donating blood was incorporated into the study by including the phenotypes of first and second degree relatives and integrating over their possible genotypes. This integration is performed without the genotypes being kept in storage.

All sample identifiers were encrypted in accordance with the regulations of the Icelandic Data Protection Authority. Approval for these studies was provided by the National Bioethics Committee and the Icelandic Data Protection Authority.

### The Icelandic genealogy

The Icelandic genealogical database contains 819,410 individuals back to 740 AD. Of the 471,284 Icelanders recorded to have been born in the 20th century, 91.1% had a recorded father and 93.7% had a recorded mother in the database. Similarly, of the 183,896 Icelanders recorded to have been born in the 19th century, 97.5% had a recorded father and 97.8% had a recorded mother.

The Icelandic genealogy was extract from many sources. Primarily from church books, censuses, the Registers Iceland (http://skra.is), local records of inhabitants and other official documents, but also from other sources such as old manuscripts, letters, annals, books of Althingi, books of judgments, books of family pedigrees, registers of farmers, registers of professional and lists of descendants.

The church books and several censuses have been computerized. The genealogical database was primarily based on the censuses of 1703, 1801 and 1910, but other censuses that have been computerized are from: 1729, 1785, 1816, 1835, 1845, 1860, 1870, 1880, 1890, 1901 and 1930.

### Illumina SNP chip genotyping

The chip-typed samples were assayed with the Illumina HumanHap300, HumanCNV370, HumanHap610, HumanHap1M, HumanHap660, Omni-1, Omni 2.5 or Omni Express bead chips at deCODE genetics. Chip SNPs were excluded if they had (i) yield less than 95%, (ii) minor allele frequency (MAF) less than 1% in the population or (iii) significant deviation from Hardy-Weinberg equilibrium (*P*<0.001), (iv) if they produced an excessive inheritance error rate (over 0.001), (v) if there was substantial difference in allele frequency between chip types (from just a single chip if that resolved all differences, but from all chips otherwise). All samples with a call rate below 97% were excluded from the analysis. The final set used for long-range phasing comprised 676,913 autosomal SNPs.

### Long range phasing

Long range phasing of all chip-genotyped individuals was performed with methods described previously^14,15^. In brief, phasing is achieved using an iterative algorithm which phases a single proband at a time given the available phasing information about everyone else that shares a long haplotype identically by state with the proband. Given the large fraction of the Icelandic population that has been chip-typed, accurate long range phasing is available genome-wide for all chip-typed Icelanders. For long range phased haplotype association analysis, we then partitioned the genome into non-overlapping fixed 0.3 cM bins. Within each bin, we observed the haplotype diversity described by the combination of all chip-typed markers in the bin.

### Whole-genome sequencing sample preparation

Paired-end libraries for sequencing were prepared according to the manufacturer's instructions (Illumina, TruSeq™). In short, approximately 1 μg of genomic DNA, isolated from frozen blood samples, was fragmented to a mean target size of 300 bp using a Covaris E210 instrument. The resulting fragmented DNA was end repaired using T4 and Klenow polymerases and T4 polynucleotide kinase with 10 mM dNTP followed by addition of an 'A' base at the ends using Klenow exo fragment (3′ to 5′-exo minus) and dATP (1 mM). Sequencing adaptors containing 'T' overhangs were ligated to the DNA products followed by agarose (2%) gel electrophoresis. Fragments of about 400–500 bp were isolated from the gels (QIAGEN Gel Extraction Kit), and the adaptor-modified DNA fragments were PCR enriched for ten cycles using Phusion DNA polymerase (Finnzymes Oy) and a PCR primer cocktail (Illumina). Enriched libraries were further purified using AMPure XP beads (Beckman-Coulter). The quality and concentration of the libraries were assessed with the Agilent 2100 Bioanalyzer using the DNA 1000 LabChip (Agilent). Barcoded libraries were stored at −20 °C. All steps in the workflow were monitored using an in-house laboratory information management system with barcode tracking of all samples and reagents.

### Whole-genome sequencing

Template DNA fragments were hybridized to the surface of flow cells (GA PE cluster kit (v2) or HiSeq PE cluster kits (v2.5 or v3)) and amplified to form clusters using the Illumina cBot. In brief, DNA (2.5–12 pM) was denatured, followed by hybridization to grafted adaptors on the flow cell. Isothermal bridge amplification using Phusion polymerase was then followed by linearization of the bridged DNA, denaturation, blocking of 3′ ends and hybridization of the sequencing primer. Sequencing-by-synthesis (SBS) was performed on Illumina GAII_x_ and/or HiSeq 2000 instruments. Paired-end libraries were sequenced at 2×101 (HiSeq) or 2×120 (GAII_x_) cycles of incorporation and imaging using the appropriate TruSeq™ SBS kits. Each library or sample was initially run on a single GAII_x_ lane for QC validation followed by further sequencing on either GAII_x_ (≥4 lanes) or HiSeq (≥1 lane) with targeted raw cluster densities of 500–800 k mm^−2^, depending on the version of the data imaging and analysis packages (SCS2.6-2-9/RTA1.6–1.9, HCS1.3.8–1.4.8/RTA1.10.36–1.12.4.2). Real-time analysis involved conversion of image data to base-calling in real-time.

### Whole-genome alignment

Reads were aligned to NCBI Build 36 (hg18) of the human reference sequence using Burrows-Wheeler Aligner (BWA) 0.5.7–0.5.9 (ref. 16). Alignments were merged into a single BAM file and marked for duplicates using Picard 1.55 (http://picard.sourceforge.net/). Only non-duplicate reads were used for the downstream analyses. Resulting BAM files were realigned and recalibrated using GATK version 1.2–29-g0acaf2d^8,17^.

### Whole-genome SNP and INDEL calling

Multi-sample calling was performed with GATK version 2.3.9 using all the 2,636 BAM files together.

Genotype calls made solely on the basis of next generation sequence data yield errors at a rate that decreases as a function of sequencing depth. Thus, for example, if sequence reads at a heterozygous SNP position carry one copy of the alternative allele and seven copies of the reference allele, then without further information the genotype would be called homozygous for the reference allele. To minimize the number of such errors, we used information about haplotype sharing, taking advantage of the fact that all the sequenced individuals had also been chip-typed and long range phased (Fig. 2)^9^. Extending the previous example, if the individual shares a haplotype with another who is heterozygous given his sequence reads, then the ambiguous individual would be called as heterozygous. Conversely, if the individual shares both his haplotypes with others who are homozygous for the major allele his genotype would be called homozygous. In order to improve genotype quality and to phase the sequencing genotypes, an iterative algorithm based on the IMPUTE HMM model^2^ which uses the LRP haplotypes was employed. Assume a SNP with alleles 0 and 1 is being phased. We let *H* be the long range phased haplotypes of the sequenced individuals and applied the following hidden Markov model (HMM) based algorithm.

Assuming that at each marker *i* the haplotype *h* has a common ancestor with a haplotype in *H*\{*h*} and denote the variable indicating this with the latent variable *z*_*i*_∈*H*\{*h*}, the hidden variable in the HMM.

Then

$$\gamma_{h,k,i}=P\left(z_{i}=k|all\;LRP\;markers\right),$$

for all *k*∈*H*\{*h*}. Given a haplotype *h* in *H*, *γ*_*h,k*_ are calculated simultaneously for all *k*∈*H*\{*h*} using the same HMM model as IMPUTE ^2^. Given the Markov assumptions of the HMM, the model is fully specified by emission and transition probabilities.

We define the emission probabilities of the HMM at each marker *i* as:

$$P\left(z_{i}=k|marker\;i\right)=\{\begin{array}{l} 1-\lambda ,\;\mathrm{if}\;h\;\mathrm{and}\;k\;\mathrm{match}\;\mathrm{at}\;i\\ \lambda ,\;\mathrm{if}\;h\;\mathrm{and}\;k\;\mathrm{mismatch}\;\mathrm{at}\;i \end{array}$$

where λ can be thought of as a penalty for a mismatch. We used *λ*=10^−7^ in our implementation. We define the transmission probabilities of the HMM model as:

$$P\left(z_{i}|z_{i-1},markers\;1,\ldots ,i-1\right)=\{\begin{array}{l} e^{-\frac{\rho_{i}}{N}}+\frac{1-e^{-\frac{\rho_{i}}{N}}}{N},\;\mathrm{if}\;z_{i}=z_{i-1}\\ \frac{1-e^{-\frac{\rho_{i}}{N}}}{N},\;\mathrm{if}\;z_{i}\neq z_{i-1} \end{array}$$

Where *N* is the number of haplotypes in *k*∈*H*\{*h*}, which for autosomal chromosomes is 2(2,636–1) here and *ρ*_*i*_=4*N*_*e*_ *r*_*i*_, where *r*_*i*_ is the genetic distance between markers *i*−1 and *i* according to the most recent version of the deCODE genetic map^18^ and *N*_*e*_ was originally meant to be an estimate of the effective number of haplotypes in the population that our sample comes from, we used *N*_*e*_=7.000. These definitions fully specify the probability distribution *P*(*z*_*i*_|*all markere*). Calculating *γ*_*h,k*_ for a single haplotype requires *O*(*MN*) operations, where *N* is the number of haplotypes and *M* is the number of markers. Since these calculations can be performed for one haplotype at a time, the calculations can be parallelized across a computer cluster for efficiency. In practice most of the *γ*_*h,k*_ will be close to zero and can be safely ignored (we used a threshold of 10^−6^ of the largest value at each marker for each *h*) greatly reducing storage requirements.

Now we are set to describe an iterative algorithm for the actual phasing. For every *h* in *H*, initialize the parameter *θ*_*h*_, which specifies how likely the one allele of the SNP is to occur on the background of *h* from the genotype likelihoods obtained from sequencing. The genotype likelihood *L*_*g*_ is the probability of the observed sequencing data at the SNP for a given individual assuming *g* is the true genotype at the SNP. If *L*_0_, *L*_1_ and *L*_2_ are the likelihoods of the genotypes 0, 1 and 2 in the individual that carries *h*, then set *θ*_*h*_:

$$\theta_{h}=\frac{L_{2}+\frac{1}{2}L_{1}}{L_{2}+L_{1}+L_{0}}.$$

For every pair of haplotypes *h* and *k* in *H* that are carried by the same individual, use the other haplotypes in *H* to predict the genotype of the SNP on the backgrounds of *h* and *k*:

$$\tau_{h}=\sum_{l\in H\setminus \left\{h\right\}}\gamma_{h,l}\theta_{l}\;\mathrm{and}$$

$$\;\tau_{k}=\sum_{l\in H\setminus \left\{k\right\}}\gamma_{k,l}\theta_{l}$$

Combining these predictions with the genotype likelihoods from sequencing gives un-normalized updated phased genotype probabilities:

$$P_{00}=\left(1-\tau_{h}\right)\left(1-\tau_{k}\right)L_{0},$$

$$P_{10}=\tau_{h}\left(1-\tau_{k}\right)\frac{1}{2}L_{1},$$

$$P_{01}=(1-\tau_{h})\tau_{k}\frac{1}{2}L_{1},$$

$$\mathrm{and}\;P_{11}=\tau_{h}\tau_{k}L_{2}.$$

Now use these values to update *θ*_*h*_ and *θ*_*k*_ to:

$$\theta_{h}=\frac{P_{10}+P_{11}}{P_{00}+P_{01}+P_{10}+P_{11}}\;\mathrm{and}$$

$$\theta_{k}=\frac{P_{01}+P_{11}}{P_{00}+P_{01}+P_{10}+P_{11}}.$$

Iterate until the maximum difference between iterations is less than a convergence threshold *ε*. We used *ε*=10^−7^.

### Whole-genome variant quality filtering

The variants identified by GATK were filtered using thresholds on GATK variant call annotations (Fig. 2). SNPs were discarded if at least one of the following thresholds for their GATK call annotation parameter was violated: QD (Variant confidence/quality by depth)<2.0, MQ (RMS mapping quality)<40.0, FS (Fisher strand)>60.0, HaploTypeScore>13.0, MQRankSum<−12.5, and ReadPosRankSum< −8.0. Indels were discarded if at least one of the inequalities QD<2.0, FS >200.0, or ReadPosRankSum<−20.0 were satisfied. The thresholds for these parameters were adopted from GATK Best Practices. In addition, SNPs and indels were discarded if one of the following thresholds were violated: DP (sequencing coverage/depth)>110,000, AN (total number of alleles in called genotypes)<4,200, and HW (Hardy-Weinberg P among sequenced samples)<10^−7^, SI (genotype information among sequenced individuals)>1.4, or if SI<0.6 for SNPs and SI<0.9 for indels. The GATK call annotation AN corresponds to the total number of chromosomes in called genotypes which equals 2×2,636=5,272 when all chromosomes can be called. The additional filtering removes 2% of the remaining SNPs but 50% of the remaining indels. It is primarily the SI condition that removes indels, which usually indicates that the failing indel is not called with a high degree of certainty and that its calling cannot be resolved in a coherent manner based on haplotype sharing.

Simple repeat regions were defined by combining the entire Simple Tandem Repeats by TRF track in UCSC hg18 with all homopolymer regions in hg18 of length 6 bp or more^19^. Variants called in these regions were ignored in the analysis.

### Liftover between hg18 and hg19

Coordinates of variants and regions were converted between hg18 and hg19 using the liftOver tool from UCSC^20^.

### Gene and variant annotation

Variants were annotated with information from Ensembl release 72 using Variant Effect Predictor (VEP) version 2.8 (refs 11,21). Only protein coding transcripts from RefSeq Release 56 (ref. 10) were considered. Transcripts occurring both in RefSeq and Ensembl were for which the coding parts of the alignments were not identical, the RefSeq functional annotations were replaced by the corresponding Ensembl annotations, based on claims by Ensembl that their alignments are more accurate and our verification of this. Variants were annotated with the classification categories of impact LoF, MODERATE, LOW, and OTHER based on the Sequence Ontology (SO)^12^ annotation from VEP. Sequence variants that could be assigned to more than one category (primarily due to their impact on more than one gene transcript) were assigned to the most severe of the applicable categories.

### Determination of ancestral state

The inference of ancestral states for SNPs and indels was based on the Ensembl Compara ancestral sequences for Homo sapiens (GRCh37) corresponding to release 65 of Ensembl^22^. These sequences are created using the Enredo-Pecan-Ortheus (EPO) pipeline^23,24^ for doing multiple sequence alignment and inference of ancestor alignments using sequences from six primates: human (Homo sapiens, GRCh37), chimp (Pan troglodytes, CHIMP2.1.5), gorilla (Gorilla gorilla, gorGor3.1), orangutan (Pongo abelii, PPYG2), macaque (Macaca mulatta, MMUL_1), and common marmoset (Callithrix jacchus, C_jacchus3.2.1). The emf2maf parser available with the Ensembl Compara API was used to convert the Compara EMF files storing the multiple alignments to MAF format. For polarization of indels, ancestral sequences were retrieved from the MAF files using bx-python.

Ancestral states of SNPs were inferred by comparison with the FASTA sequence for the Ensembl Compara human ancestor sequence for Homo sapiens (GRCh37).

When infering the ancestral state of an indel based on the Ensembl Compara multiple alignments and inferred ancestor alignments for the six primates, we considered the ancestral state closest to Homo sapiens in the phylogenetic tree in a window spanning 5 bp to the left and right of the indel and assignment position plus the indel length; the polarization state was left undetermined for indels in regions where an ancestral state was not available from the Ensembl Compara data. We compared the difference in the number of bases in the aligned sequences in the window to find out whether there was an evidence of an indel in the ancestral sequence. In cases where there was no difference in the number of bases between ancestral sequence and the sequence for Homo sapiens, the ancestral state was said to be ‘derived’ (that is, the Homo sapiens reference allele is the same as the ancestral allele) if there was at most one mismatch in the sequences in the window; if there were more than one mismatch in the sequence, the state was left undetermined. When the difference in the number of bases was equal to the indel length the most likely ancestral state was only designated as ‘ancestral’ (that is, the Homo sapiens alternative allele is the same as the ancestral allele) if at most one mismatch was in the sequence in the window to the left and right of the indel, where, in the case of insertions, we also required the insertion bases to match the insert sequence exactly. In all other cases the ancestral state was left undetermined.

### Estimation of the transition/transversion ratio

Transition/transversion (Ts/Tv) ratios were calculated for various sets of identified SNPs. SNPs with alleles is A and G, or C and T, were caused by transition mutations and all other SNPs by transversions. The Ts/Tv ratio within a set of SNPs was estimated as the number of SNPs caused by transitions divided by the number of SNPs caused by transversions.

### Overlap with ESP and dbSNP

We assessed the overlap between the variants we discovered in Iceland with those in dbSNP (build 137)^25^ and with those reported by the ESP^14,15^. The Icelandic variants were counted as existing in the ESP or dbSNP if a variant at the same position and with the same allele was present in the database.

## Data Records

List of 21 million sequence variants identified in 2,636 whole genome sequenced Icelanders is accessible at the European Variant Archive (EVA) (Data Citation 1). The data for each sequence variant consists of the chromosome and position (in NCBI builds 36 (hg18), 37 (hg19) and 38 (hg38)), reference allele, alternative allele, non-reference allele frequency, the maximal consequence VEP annotation, the affected gene, the HGVSc coding sequence names, the HGVSp protein sequence names, the impact class and the dbSNP rsIDs from build 142.

## Technical Validation

**Figure 3:**
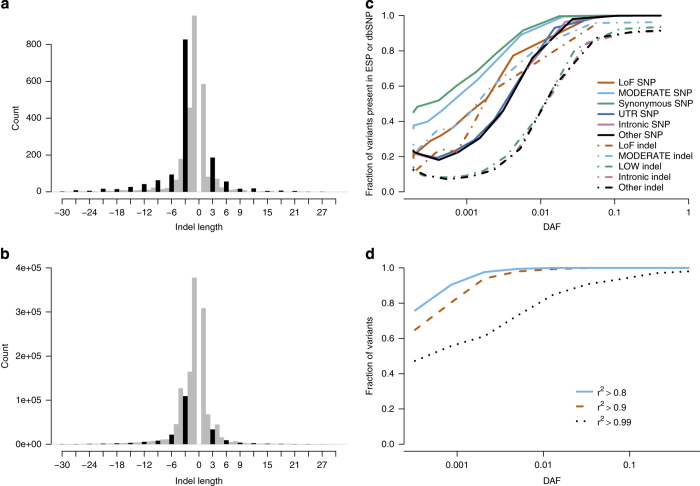
Validation of sequencing data. Distribution of indel length inside (**a**) and outside (**b**) protein coding regions. The 4,001 indels inside protein coding regions. Insertions have a positive length and deletions have a negative length. Indels that are not multiples of three are colored grey. Indels that are a multiple of three are colored black. The fraction of SNPs and indels identified in 2,636 Icelanders present in dbSNP (build 137) or the Exome Sequencing Project (ESP) by consequence (**c**). The analysis was restricted to 16,587,813 SNPs and 1,191,089 indels for which the ancestral allele could be inferred. Shown is the overlap with the union of dbSNP and ESP as a function of derived allele frequency (DAF) by annotation and variant type. Comparison of imputed and chip genotypes (**d**). Shown is the fraction of the 28,204 SNPs identified in exons and splice regions and present on SNP chips that have r^2^>0.8, 0.9 and 0.99 between imputed and chip genotypes as a function of their derived allele frequency (DAF). Figure reproduced from Figs 1, 5 and Supplementary Fig. 5 of ref. 13.

As a measure of quality of our SNP data we compared the transition/transversion ratio (Ts/Tv) to previously reported ones (Table 4). Intergenic regions have a Ts/Tv ratio of 2.13. Synonymous SNPs have the highest Ts/Tv ratio (5.42), apart from stop retained variants which are obligate transitions. Our Ts/Tv ratio estimates are similar to those reported by the ESP based on Illumina data^5^ and those reported by the Huvariome resource based on sequencing by Complete Genomics^26^. Similarly, we examined the distribution of indels in coding regions and observed a clear deficit of indels that shift the reading frame (frameshift indels) (Fig. 3a,b).

A comparison of our variants to those in dbSNP^25^ and the ESP^14,15^ revealed that essentially all common SNPs found in Iceland have been recorded in dbSNP (99.8% of SNPs with DAF>2%) (Fig. 3c). Indels are more difficult to call than SNPs and their position can be ambiguous. A substantial fraction of the common indels found in Iceland is not present in either dbSNP or ESP (Fig. 3c). Few of the rare indels found in Iceland have been recorded in dbSNP. In coding regions, SNPs and indels with DAF<0.5% have a 60 and 20% chance of being present in at least one of the databases, respectively.

The 104,220 long range phased of Icelanders genotyped for 676,913 autosomal SNPs using Illumina chips (Fig. 3d) provide an opportunity for validating SNP predictions. Of variants with MAF over 0.1%, 99.5% were imputed with information over 0.8. The concordance between 28,204 chip typed SNPs, that were not part of the long range phasing set, was high (98.4% of SNPs with DAF>1% were imputed accurately (r^2^>0.9)).

To further assess the quality of the variant calling we Sanger sequenced individuals predicted to be homozygous for a set of rare LoF variants, enriched for indels over SNPs. Genotypes were observed for 47 out of 49 LoF variants (96%). Out of the 140 individuals imputed to be homozygous for one of these 47 variants yielding genotypes, genotypes were obtained for 134 (96%), 128 of which were homozygotes (96%).

## Usage Notes

Icelandic law and the regulations of the Icelandic Data Protection Authority prohibit the release of individual level and personally identifying data. We are actively participating in multiple meta-analysis based on our data and are in collaboration with groups at over 100 international universities and institutions. Therefore, collaborations based on our sequencing data are based on the release of summary level statistics, such as effect sizes and *P*-values for meta-analysis, or the collaborators travelling to our Icelandic facilities for local data access.

## Additional information

**How to cite this article:** Gudbjartsson, D. F. *et al.* Sequence variants from whole genome sequencing a large group of Icelanders. *Sci. Data* 2:150011 doi: 10.1038/sdata.2015.11 (2015).

## Supplementary Material



## Figures and Tables

**Table 1:** The 50 most prevalent conditions among the 2,636 sequenced Icelanders.

**Disease**	***N***
Coronary Artery Disease	474
Chronic Kidney Disease	424
Obesity	394
Hypertension	371
Type 2 diabetes	307
Osteoporosis	260
Atrial Fibrillation	259
Myocardial Infarction	246
Alzheimer's disease	214
Asthma	211
Osteoarthritis	209
Urinary Tract Infection	208
Systemic Lupus Erythematous	207
Alcohol Dependence	199
Breast Cancer	196
Depression	171
Sleep Apnea	165
Autism Spectrum Disorders	160
Kidney Stones	160
Nicotine Dependence	158
Prostate Cancer	158
Gallstones	156
Schizophrenia	155
Attention Deficit Hyperactivity Disorder	154
Glaucoma	138
Mental Retardation	138
Colorectal Adenoma	134
Migraine	132
Age Related Macular Degeneration	127
Ischaemic Stroke	122
Psoriasis	120
Epilepsy	118
Tuberculosis	118
Basal Cell Carcinoma Of The Skin	115
Parkinson's Disease	108
Diverticular Disease	107
Cataract	106
Bronchitis	98
Tourette Syndrome	84
Heart Failure	81
Emphysema	80
Dyslexia	79
Hypothyroidism	79
Sick Sinus Syndrome	78
Peripheral Artery Disease	77
Rheumatoid Arthritis	73
Benign Prostatic Hyperplasia	70
Congenital Heart Disease	70
Hypertension In Pregnancy	69
Panic Disorder	69
Table reproduced from Supplementary Table 1 of ref. 13.	

**Table 2:** The demographics of the sequenced, chip-typed and relatives of chip-typed individuals.

**Demographic**	**Sequenced**	**Chip-typed**	**Relatives of chip-typed**
N	2,636	104,220	294,212
Female (%)	54.0	55.1	46.8
YOB* (s.d.)	1950 (23)	1953 (23)	1964 (36)
Alive†(%)	72.5	84.9	72.9
Age‡ (s.d.)	55 (20)	56 (70)	33 (20)
Lifespan§ (s.d.)	79 (13)	80 (13)	59 (28)

*Year of birth.

^†^Fraction currently alive.

^‡^Current age for the living.

^§^Age at death. Table reproduced from Supplementary Table 2 of ref. 13.

**Table 3:** The number of indels and SNPs by impact group and minor allele frequency (MAF).

**Type**	**MAF**	**LoF** * **(Frameshift indels, splice acceptor/ donor, stop gained/lost, initiator codon)**	**MODERATE** **(Inframe indels, missense, splice region)**	**LOW** **(Synonymous, stop retained, 3′/5′ UTR)**	**Other** **(Intronic, intergenic)**	**Total**
SNP	≥0.5%	602 (0.0070%)	36,282 (0.42%)	108,850 (1.3%)	8,445,855 (98.3%)	8,591,589
	0.1% to 0.5%	915 (0.023%)	29,659 (0.76%)	59,076 (1.5%)	3,836,528 (97.7%)	3,926,178
	<0.1%	2,462 (0.034%)	57,209 (0.80%)	101,751 (1.4%)	7,010,453 (97.7%)	7,171,875
	All	3,979 (0.020%)	123,150 (0.63%)	269,677 (1.4%)	19,292,836 (98.0%)	19,689,642
Indel	≥0.5%	418 (0.0609%)	797 (0.12%)	8,352 (1.2%)	676,820 (98.6%)	686,387
	0.1% to 0.5%	677 (0.229%)	568 (0.19%)	4,380 (1.5%)	290,492 (98.1%)	296,117
	<0.1%	1,721 (0.375%)	1,027 (0.22%)	6,757 (1.5%)	449,563 (97.9%)	459,068
	All	2,816 (0.195%)	2,392 (0.17%)	19,489 (1.4%)	1,416,875 (98.3%)	1,441,572
Percentages are for the proportion of variants that fall in each class within the row.						

*Loss of function. Table reproduced from Table 1 of ref. 13.

**Table 4:** The transition/transversion (Ts/Tv) ratio by SNP consequence.

**Consequence**	***N***	**Ts/Tv**			
		**Iceland**	**Tennessen** ***et al.*** ^5^	**Stubbs** ***et al.*** ^26^	**Impact**
splice acceptor	539	1.59	1.69	2.42	LoF
splice donor	783	1.86		2.81	LoF
stop gained	2,269	2.15	2.13	1.93	LoF
stop lost	100	0.89			LoF
initiator codon	269	2.16			LoF
missense	108,568	2.36	2.31	2.14	MODERATE
splice region	14,107	2.90			MODERATE
synonymous	71,215	5.42	5.60	5.20	LOW
stop retained	68	Inf*			LOW
3′UTR	164,852	2.39		2.15	LOW
5′UTR	32,275	1.72			LOW
intronic	7,299,496	2.29		2.22	OTHER
downstream of gene	422,534	2.33			OTHER
upstream of gene	493,094	2.12			OTHER
Other intergenic	10,950,712	2.13		2.07	OTHER
The Icelandic ratios are based on the SNPs found in the 2,636 whole-genome sequenced Icelanders and compared to previously reported ratios^5,26^.					

*The Ts/Tv ratio is infinite for the stop retained class by definition. Table reproduced from Supplementary Table 4 of ref. 13.
